# Evaluation of pitting corrosion by dynamic speckle pattern analysis

**DOI:** 10.1038/s41598-023-35559-w

**Published:** 2023-05-26

**Authors:** Omid Pedram, Ramin Jamali, Vahid Abbasian, Vahideh Farzam Rad, Arash Darafsheh, Ramin Khamedi, Esmaeil Poursaeidi, Ali-Reza Moradi

**Affiliations:** 1 Department of Mechanical Engineering, University of Zanjan, Zanjan, 45371-38791 Iran; 2 Department of Physics, Institute for Advanced Studies in Basic Sciences (IASBS), Zanjan, 45137-66731 Iran; 3 Imaging Science Program, McKelvey School of Engineering, Washington University in St. Louis, St. Louis, MO 63130 USA; 4 Department of Radiation Oncology, Washington University School of Medicine in St. Louis, St. Louis, MO 63110 USA; 5 Department of Mechanical Engineering, School of Engineering & Applied Science, Khazar University, Baku, AZ1096 Azerbaijan; 6 School of Nano Science, Institute for Research in Fundamental Sciences (IPM), Tehran, 19395-5531 Iran

**Keywords:** Imaging and sensing, Mechanical engineering

## Abstract

There is an increasing interest in non-destructive and real-time high-resolution approaches for corrosion studies in metals. In this paper, we propose the dynamic speckle pattern method as a low-cost, easy-to-implement, and *quasi in-situ* optical technique for the quantitative evaluation of pitting corrosion. This type of corrosion occurs in a specific area of a metallic structure and causes holes formation leading to structural failure. A Custom 450 stainless steel sample, placed in 3.5 wt% NaCl solution and applied to a $350 \,\hbox {mV}_{SCE}$ potential to initiate the corrosion, is used as the sample. The speckle patterns formed by the scattering of a He-Ne laser light is changed over time due to any corrosion in the sample. The analysis of the time-integrate speckle pattern suggests that the growth rate of pitting decreases with time.

## Introduction

The chloride ion ($\hbox {Cl}^-$) adsorption process may change the ion conductivity of passive films; as a result, some metals are prone to pit formation. Due to hydrolysis inside the pit, chloride ions moved into the pit to achieve electrical neutrality, are adsorbed on the pit surface, leading to pitting propagation^1^. Once a corrosive environment is combined with a stressconcentrating environment, stress corrosion cracking (SCC) may occur. Characterization of the progression of the damage is essential for increasing safety and minimizing the economic cost of the system^2^. Experimental methods, such as eddy current, electrochemical measurements^3^, optical microscopy, scanning electron microscopy (SEM), x-ray diffraction^4–6^, atomic force microscopy (AFM)^7^, and digital holography^8^ can provide useful information about pitting corrosion. When a coherent light illuminates a rough sample, a dynamic speckle pattern forms such that any movement in either the exterior surface or interior structure of the sample under study can change that over time. The statistical analysis of such speckle patterns provides important information about the dynamic sample^9,10^.

There is an increasing interest in using dynamic speckle pattern method in life and material sciences^11–13^. To several applications, the method has been used for blood flow monitoring^11^, polymer surface characterization^14,15^, seed^16^ and fruit analysis^17^, parasite activity evaluation^18^, bone scaffold analysis^19^, examination of the paints drying^20^, detecting imperfections of the subsurface in multilayer composites^21^. The technique has also shown promising results in corrosion studies, such as applying electronic speckle pattern interferometry (ESPI) and digital speckle correlation (DSC), for the detection of corrosion, pitting, and crevice corrosion^22,23^. Fricke-Begemann et al.^24^ examined the micro-topography changes of a metal surface during a corrosion process using decorrelation of scattered speckle fields. Moreover, surface corrosion processes of an iron (Fe) immersed in sulfuric acid was studied utilizing digital speckle pattern interferometry (DSPI) by Andrés et al.^25^.

In this paper, we introduce utilization of dynamic speckle pattern approach for quantitative assessment of pitting corrosion in a metallic sample. In order to prove the capability and efficacy of the proposed method, dynamic speckle patterns analysis is performed for the real-time monitoring of pitting corrosion in a Custom 450 stainless steel sample. The significant feature of this work is introducing a simple *quasi in-situ* approach for quantitative evaluation of corrosion, which may not be achieved by conventional microscopy systems.

## Materials and methods

### Sample preparation

A piece of Custom 450 stainless steel was harvested from a frame-type gas turbine installed in a seaside power plant, specifically, from the hub part of a failed blade which was in the first stage of the compressor blade. A wire electrical discharge machine was used to cut the sample into a desired dimension (0.5$\times 74\times$5 $\hbox {mm}^3$). The mechanical and corrosion properties of the sample material have been described elsewhere^4,26,27^. The sample was mechanically abraded by a series of wet silicon carbide sandpapers with grit sizes of #100, #220, #400, #600, #800, #1000, #2000, and #3000. Then, it was polished with a 2.5 $\mu$m alumina solution to achieve a mirror-like smoothness before it was cleaned by alcohol.

### Electrochemical test

The NaCl electrolyte (3.5 wt%) was prepared from reagents of analytical grade and twice-distilled water. Potentiostatic and potentiodynamic electrochemical tests were carried out to obtain the pitting corrosion, as well as its time and potential, according to ASTM G5^28,29^. All electrochemical measurements were performed using an OrigaFlex multi-channel system at room temperature (25 $\pm \,\, 1\,\,^{\circ }$C) under non-deaerated condition. Tests were conducted using a three-electrode electrochemical cell with a saturated calomel electrode (SCE) as a reference, platinum as an auxiliary electrode, and a Custom 450 stainless steel strip with an area of 4 $\hbox {mm}^2$ as the working electrode. All applied potentials were measured versus $\hbox {V}_{{SCE}}$. At least five experiments were carried out for each typical test.

**Figure 1:**
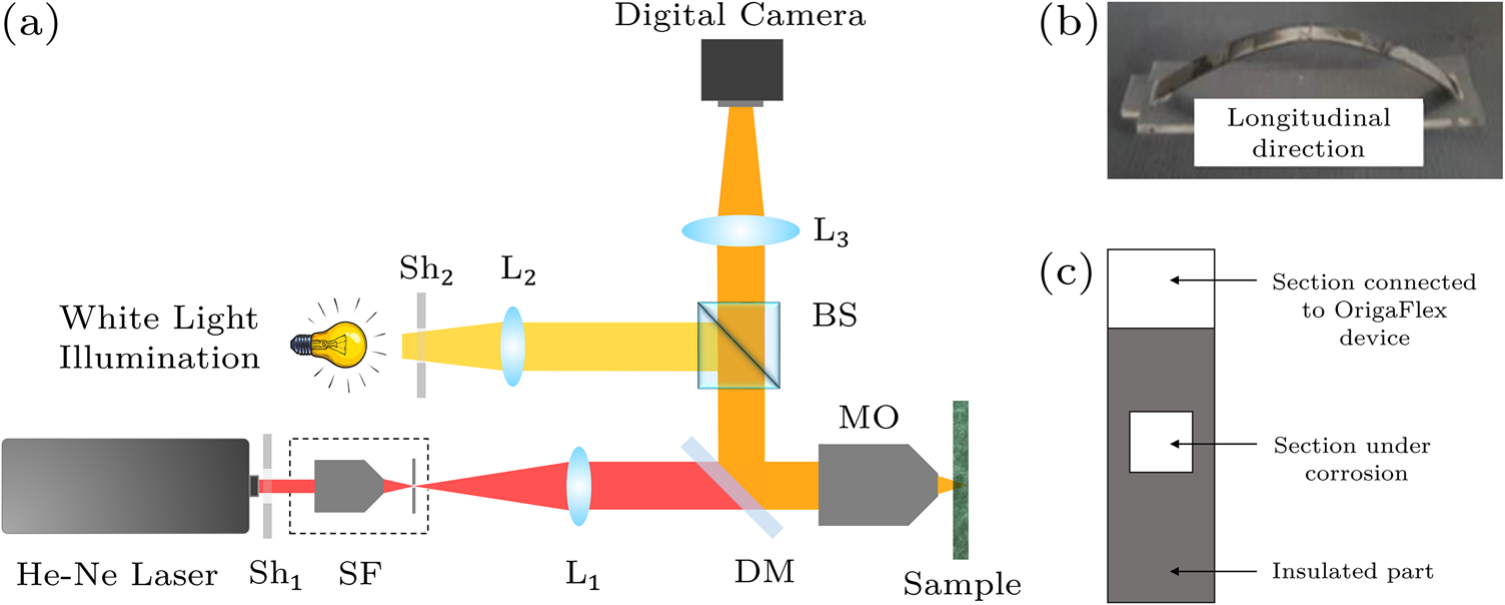
(**a**) Experimental arrangement used for dynamic speckle pattern and bright filed microscopy; SF, spatial filter; $\hbox {L}_1$ & $\hbox {L}_2$, lens; $\hbox {L}_3$, collecting lens; DM, dichroic mirror; BS, beam splitter and Sh, shutter. (**b**) Two point bending sample. (**c**) Sample schematic under electrochemical test.

### Experimental procedure

Figure 1a schematically shows the experimental arrangement used for recording dynamic speckle patterns. A He-Ne laser beam (632.8 nm, 5 mW) was passed through a spatial filter (SF), removing unwanted spatial frequencies in the Fourier space through a pinhole located in the lens’ focal plane. The resultant diverging laser beam was then collimated using a lens, $\hbox {L}_{{1}}$, (100 mm focal length, + 10.0 D). The beam passed through a dichroic mirror (DM) and then focused onto the sample by a microscope objective (MO) (5$\times$, NA = 0.14, 34.0 mm working distance). The backscattered light as speckle patterns was collected by the MO and reflected toward a digital camera by the DM. The light passed through a beam splitter (BS) module and guided by lens, $\hbox {L}_{{3}}$, (50 mm focal length, + 20.0 D) to the camera. A digital CMOS camera (EOS 1200D, Canon, TTL-CT-SIR, 18.7 megapixels) was used for recording images at 1280$\times$720 resolution with an exposure time of 0.20 ms at 50 fpm. The speckle setup was integrated with a conventional microscope through integrating a white light source using the beam splitter. The white light was collimated by lens, $\hbox {L}_{{2}}$, (115 mm focal length, + 8.7 D) and directed to the sample by BS, DM, and MO. The reflected beam from the sample was collected by the MO and followed the same imaging path toward the camera in order to obtain micrographs of the sample. A shutter was placed after each light source to control when the light from each source should reach the sample. During the recording speckle pattern data, the shutter $\hbox {Sh}_{{2}}$ was closed to stop the white light reaching the sample. Similarly, the shutter $\hbox {Sh}_{{1}}$ was closed during recording of micrographs. A wire-cut electrical discharge machine was utilized to prepare the test samples shown in Fig. 1b. Custom 450 is sensitive to pitting corrosion in 3.5 wt% NaCl solution at the potential of 350 $\hbox {mV}_{{SCE}}$, as already described^3^. The experiments were designed for the real-time monitoring of pitting corrosion at the maximum bending region. To this end, a potential of 350 $\hbox {mV}_{{SCE}}$ was applied to the sample for 30 min (Fig. 1c). The monitoring was performed by recording the scattered laser light from the sample under corrosion, i.e. the speckle patterns. The dynamic speckle patterns associated with the pitting propagation were analyzed at time intervals of 10, 20, and 30 min after the process was started. The evaluation of the pitting corrosion was performed by numerical processing of the recorded speckle patterns.

In order to prevent corrosion of the part connected to the working electrode and ensure proper placement of device connections in the circuit, the part must be kept out of the solution during testing. This means that the sample cannot be positioned horizontally in the solution under the microscope, as the electrode-connected part would be submerged. To maintain the part out of the solution, the sample must be viewed vertically within the solution during the testing process. To achieve this, a digital camera was connected to a horizontally placed microscope on the table, allowing the container chamber to be positioned vertically and the surface to be magnified and filmed. As a result, the surface can be examined in real-time at the point of maximum bending when the potential is applied and the corrosion process begins. The container used in the experiment is specifically designed using 3 mm thick plexiglass to ensure the sample was placed in a completely vertical position, with proximity to the observed corrosion site. In order to prevent any interference caused by the thickness of the plexiglass, a 0.1 mm lamella was used in place of the cut section. The electrode placement was installed on the lid of the container, and all sides and surfaces of the lamella were insulated using chloroform solution and aquarium glue to prevent solution leakage or movement. To obtain a clear image of the sample surface, the container was carefully re-adjusted rather than relying on microscope settings.

Dynamic speckle patterns become more pronounced when an illuminated surface includes any kind of activity. Based on the origins and characteristics of dynamic speckles the knowledge about the inner dynamics of the phenomena may be increased. The more inner dynamics of the samples are known, a better insight can be obtained in controlled experiments and simulations to assess how these dynamics show up in the speckle evolution. The advantage of the presented methodology over the common polymer characterization techniques includes possibility of dynamic and live acquisition of information about the samples. It is a nondestructive and non-contact method and provides spatio-temporal information integration. In addition, this technique is free of phototoxic effects on the sample since a very low laser power is used to illuminate the samples. In the dynamic speckle method, data can be extracted and analyzed instantly within the frame rate limit of the speckle capturing device. It means that the evolution of the sample can be obtained at any moment. The He-Ne laser possesses sufficient coherency and stability. The laser is switched on at least half an hour before the experiment to ensure intensity stability, which is of high importance in the present method. The laser is kept on during the experiments and by using a laser shutter $\hbox {Sh}_{{1}}$, without touching the elements of the setup, the beam is blocked. When required, the laser shutter $\hbox {Sh}_{{1}}$ is removed and the data is acquired. The uniformity of the beam is checked by replacing the sample by a mirror and collecting the reflected light by the camera for about a minute.

## Numerical processing

The sample activity, specifically in dynamic materials, can be expressed by various metrics. The main goal of the present work was to characterize the pitting corrosion activity as a function of time. The spatial and temporal coherence of the laser illumination makes it possible to preserve the stability of the speckle pattern for static scatterers. Hence, the observable activity in the dynamic laser speckle can be associated to internal characteristics, such as scaffold activity, growth and cell division, lipid phase separation, movement of cytoplasmic and biochemical reactions, and water-related activities^19,30–34^. The activities of the pitting corrosion can be attributed to the changes in the surface roughness. The numerical processing of recorded speckle patterns includes statistical parameters which are described in this section.

**Figure 2:**
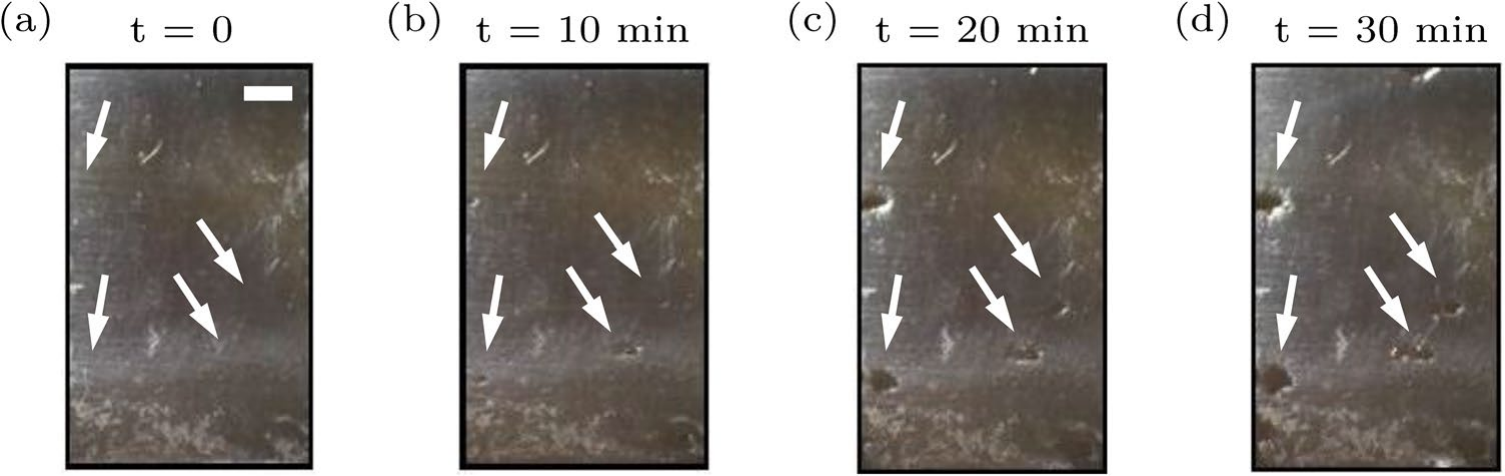
Two intermediate pits, the growth of pits on the surface at the maximum bending region at the potential of 350 $\hbox {mV}_{{SCE}}$ (**a**) before the start of test starting, (**b**) after 10 min, (**c**) after 20 min, (**d**) after 30 min. The white scale bar corresponds to $700 \,\upmu \hbox {m}$.

Activity-related measurements were performed while the pitting corrosion was initiated by applying a potential of 350 $\hbox {mV}_{{SCE}}$ to the sample. We label the aforementioned structural alternations as pitting corrosion activities. Recorded images were analyzed by converting the recorded movie to an image sequence. Numerical analysis was performed on such consecutive images to investigate the corrosion^11,35,36^. The time history speckle pattern (THSP) as a two-dimensional matrix presents M points set evolution through time in sequential speckle patterns are termed image data pack points. In the THSP matrix, the rows represent points set in a speckle pattern, and the columns represent the time. From the initial speckle pattern, the set of random M points was selected such that the first column of the THSP was reconstructed. The other THSP matrix columns were constructed by the equivalent points in consecutive patterns. In order to have the sample activity level as the graphical sign, the THSP was provided such that more line variation of the THSP presents the high activity of the sample under examination. As shown in Fig. 2b–d for 10, 20, and 30 min from starting the experiment. The THSP shows over time that the sample activity was increased and this cloudiness of the THSP occurs by increasing the corrosion pitting.

The THSP concept is basically used for numerical calculations such as co-occurrence matrix (COM), and inertia moment (IM) to name a few. Additionally, more parameters are possibly being investigated like motion history image (MHI), and roughness parameters (skewness, kurtosis, etc.) without the need for THSP^33,35,37–39^. The co-occurrence matrix (COM) can be considered as the intermediary matrix which is graphical and is used for the assessment of the consecutive pixels’ dispersion in a THSP of M points which surveys N samples. COM illustrates a histogram related to intensities evolutions:

1 $$\begin{aligned} {\mathrm{COM}}(i,j)=\sum _{m=1}^{M}\sum _{n=1}^{N-1} {\left\{ \begin{array}{ll} 1, &{} \quad \text {if THSP}(m,n) = i \\ &{} \quad \text {and THSP}(m,n+1) = j,\\ 0, &{} \quad \text {otherwise.} \end{array}\right. } \end{aligned}$$

**Figure 3:**
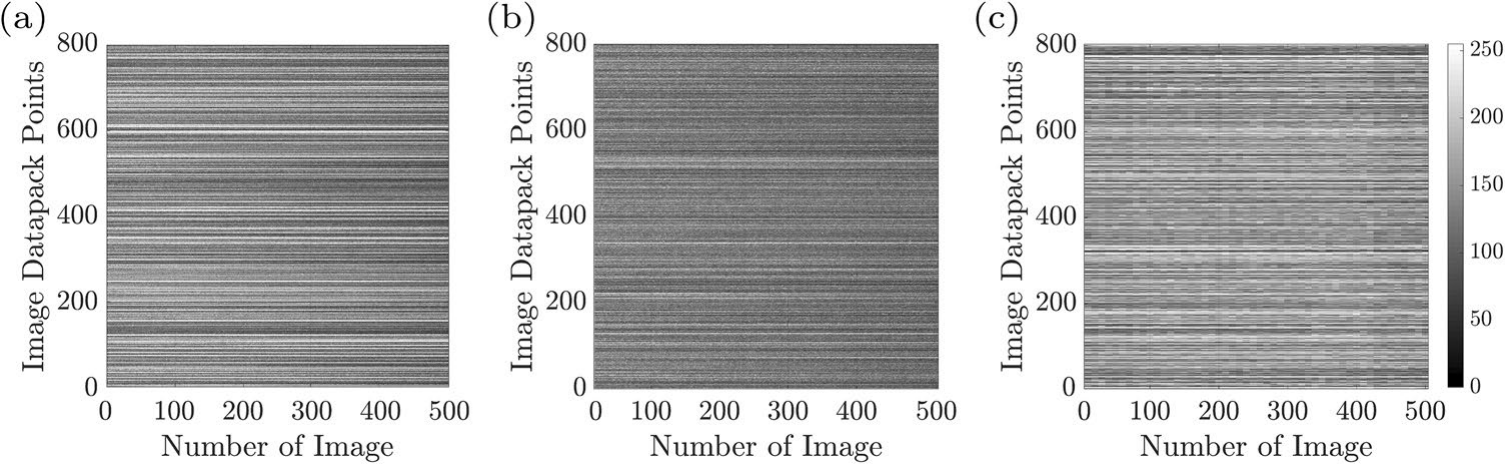
The time history speckle pattern (THSP), formed by tracking 800 random points all over the collection of 500 speckle patterns of the sample (image datapack points). The results at (**a**) $\hbox {t} = 10 \,\hbox {min}$, (**b**) $\hbox {t} = 20 \,\hbox {min}$, (**c**) $\hbox {t} = 30 \,\hbox {min}$ after the experiment starts, are shown.

**Figure 4:**
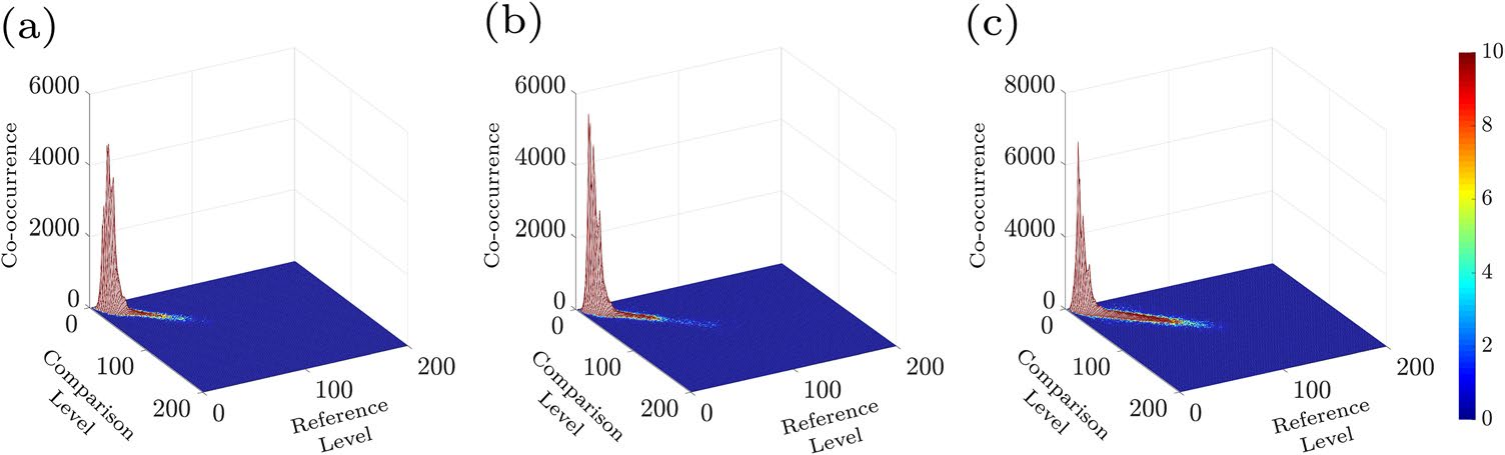
(**a**–**c**) The 3D plot of COM images of the associated time history speckle patterns (THSP) in Fig. 3 at every 10 min after the experiment started. Reference level and comparison level show intensity levels of i and j in Eq. (1), respectively.

In order to decrease the influence of the inhomogeneities in the images, a normalization was done in a manner that the total sum in every COM line was equal to 1. The number of points (M) and the number of images (N) in a row of the data pack were used to perform sample analysis. Figure 4a–c shows the COMs which are the results of samples set in different pitting corrosion states for t = 10, 20, and 30 min. Demonstration of i and j (intensity levels) is by comparison level and reference level, respectively. Moreover, structure deformation happens when points spread are observed along the origin diagonal. Lower points and less spread around original diagonal pitting corrosion represent low deformation, such as not being any pitting in starting experiments. Considering IM indicating numerical activity and the statistical outcome is done by Eq. (1):

2 $$\begin{aligned} {\mathrm{IM}}=\sum _{i}^{}\sum _{j}^{} \frac{{\mathrm{COM}}(i,j)}{\sum _{m}{\mathrm{COM}}(i,m)}|i-j|^2. \end{aligned}$$

**Figure 5:**
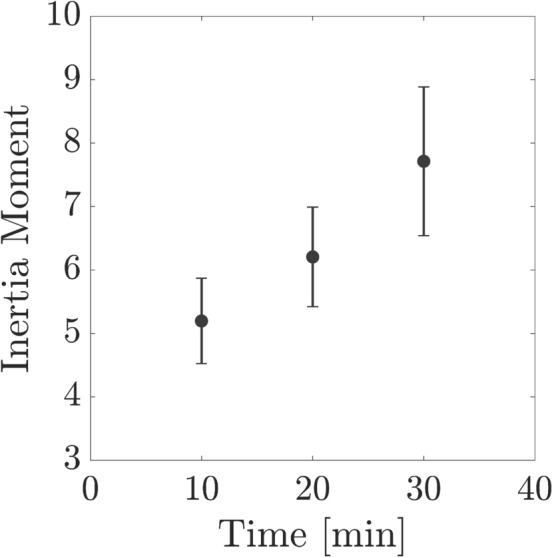
Average inertia moment over the samples’ THSPs as a function of time.

Figure 5 demonstrates the resulted IM of the samples at different times. As illustrated, in the pitting corrosion cases the IM value is moderately increased as the growth pitting is progressing. The IM increase values because of pitting corrosion evolution through times is clearly appreciable. The inertia moments name is deduced from the point view of mechanical resembling. Moreover, there are more parameters relating to the sample activity for characterizing motions in images in a row through time, such as the motion history image (MHI). The MHI is illustrative of the static image in that the intensity of pixels is related to the recent image’s consecutive motion and basically contains the information for characterizing the object’s motion while being active^40–43^. This image looks to include the essential information to determine how a surface object has evolved during the activity. This parameter is an indicator for the activity of a pixel, which has absolute intensity jump higher to *U*. This method generates the last image by means of the each pixel (i,j) following in the MHI. The comparison indicator for sequential images changes of objects is done by the below equation:

3 $$\begin{aligned} Z_k = I_k - I_{k-1}, \end{aligned}$$

where $\hbox {I}_{k-1}$ and $\hbox {I}_k$ indicates the grayscale images in the instants $k-1$ and *k*. However, the image which is resulted from the subtracted images, presented by Eq. (4) is applied:

4 $$\begin{aligned} T_k(i,j)=\left\{ \begin{array}{ll} 1 &{}\text {if }|Z_k(i,j)|>U, \\ 0 &{}\text {if }|Z_k(i,j)|\leqslant U, \end{array}\right. \end{aligned}$$

where *U* is the threshold factor and $\hbox {T}_k(i,j)$ is the threshold image at each moment *k*. Eventually, the MHI method is the threshold images weighting by a constant presenting the *NTIMES* at the *k* instant, as given in Eq. (5):

5 $$\begin{aligned} MHI = 255 \sum _{l=0}^{NTIMES-2} T_{k-l}~h_l, \end{aligned}$$

The $\hbox {h}_l$ value is given by:

6 $$\begin{aligned} h_l = \frac{NTIMES-l}{M}, \end{aligned}$$

and,

7 $$\begin{aligned} M = NTIMES(NTIMES+1)/2. \end{aligned}$$

**Figure 6:**
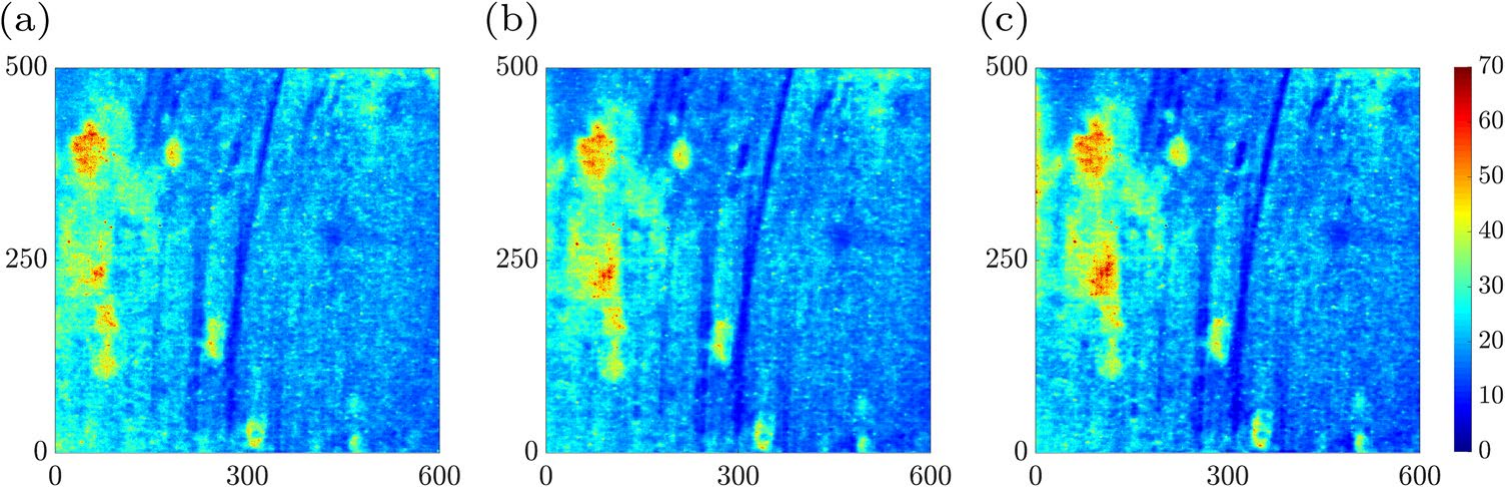
(**a**–**c**) 2D Map of activities obtained by the motion history image (MHI) on a collection of images of a sample every ten minutes after the experiment starts.

Where the $h_l$ value as the weighting parameter is based on the data images and *M* is equivalent with summation of *NTIMES* first natural numbers so that the summation of all $h_l$ is ever one. Figure 6a–c shows an image of pitting corrosion in different durations of 10, 20, 30 min, where its movement will clear understanding the MHI in relation with alternations in the image all over space.

Besides, parameters related to roughness like various moments of the intensities deviated from the mean values, in all patterns are possible to be calculated via the speckle intensity patterns through statistical processing. The expression of average intensities variations to mean value data is as ($R_{P1}$), where the standard deviation description of the distribution is the intensities root mean square ($R_{P2}$)::

8 $$\begin{aligned} R_{P1}= & {} \frac{1}{P~Q} \sum _{p=1}^{P} \sum _{q=1}^{Q}|I(p,q) - \langle {I}(p,q)\rangle |, \end{aligned}$$

9 $$\begin{aligned} R_{P2}= & {} \bigg [\frac{1}{P~Q} \sum _{p=1}^{P} \sum _{q=1}^{Q}\left[ I(p,q) - \langle {I}(p,q)\rangle \right] ^2\bigg ]^{\frac{1}{2}}, \end{aligned}$$

Where *Q* and *P* indicates the vertical and horizontal speckle pattern dimensions, *p* and *q* include the numbers of pixel counter and I as the intensity of whole the speckle patterns. The usefulness of such metrics could be providing a common estimation of distribution roughness. Equivalent, skewness $R_{P3}$, and kurtosis $R_{P4}$, can be considered another usual roughness parameters applicable for evaluating the samples structures:

10 $$\begin{aligned} R_{P3}= & {} \frac{1}{P~Q~R_{P2}^3} \sum _{p=1}^{P} \sum _{q=1}^{Q}\left[ I(p,q) - \langle {I}(p,q)\rangle \right] ^3, \end{aligned}$$

11 $$\begin{aligned} R_{P4}= & {} \frac{1}{P~Q~R_{P2}^4} \sum _{p=1}^{P} \sum _{q=1}^{Q}\left[ I(p,q) - \langle {I}(p,q)\rangle \right] ^4. \end{aligned}$$

According to the $R_{P3}$, the description being calculated as the third moment of the deviation away from the mean value is the measurement of the symmetries degree relating to the distribution of the intensities. Positive skewness demonstrates a distribution including more peaks but negative skewness shows more valleys such as low intensities^44–46^. Kurtosis $R_{P4}$ is the parameter measuring the sharpness of distribution throughout the pattern. We used skewness as the represented roughness analysis of the speckle pattern. Derivation of several parameters is possible to have more information considering the samples through their statistical analysis. Significantly, the parameters calculation of roughness related to the intensity distribution all over the speckle pattern is equivalent to the sample surface roughness. The sets of aforementioned parameters are different as are related to the height increase and decrease. The previous set is concerned with the intensity changes throughout the surface of the sample. Equivalent trends for the two different sets of parameters in various types of samples have been already examined and reported. Consequently, Skewness and kurtosis demonstrate evolutions in the pitting corrosion phenomenon through time. It is clearly shown that it increases in time because of pitting corrosion growth. As exemplary for roughness changes through time we have plotted the 2D map of skewness changes for 10, 20, and 30 min.

**Figure 7:**
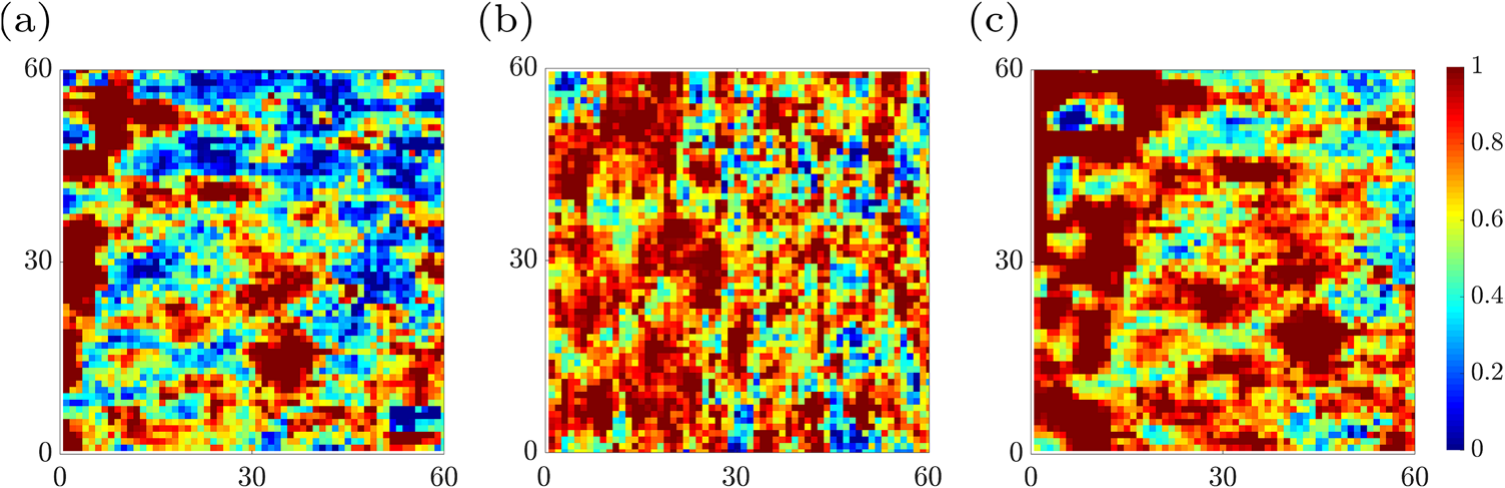
(**a**–**c**) Temporal speckle skewness matrix every ten minutes after the experiment starts.

## Results and discussion

Figure 2a–d show the formation and growth of two intermediate pits at the maximum bending region of the sample at the beginning of the experiment when the 350 $\hbox {mV}_{{SCE}}$ potential was applied, after 10 min, 20 min, and 30 min, respectively. The sample was not cleaned during the experiment. Thanks to the real-time pitting monitoring, we were able to observe that corrosion products precipitated at the bottom of the container once they came out from the pits. Due to the gravity, the vertical positioning of the sample allowed corrosion products to flow more easily out of the pits compared to horizontal positioning of the sample. The metal loss from pitting corrosion is less than that from uniform corrosion; nevertheless, because this type of corrosion occurs over a smaller area, it extends to a greater depth. Corrosion products conceal pits and generally cause devices to fail by perforation, or initiate stress corrosion cracks^47,48^.

Comparison of Fig. 2c and d in the last 10 min of the test shows formation of new pits instead of propagation of previous pits in our sample. The activities of pitting corrosion which are revealed by various parameters through the speckle patterns analysis can be attributed to the interactions between the sample and the 3.5 wt% NaCl solution at potential of 350 $\hbox {mV}_{{SCE}}$ that cause structural changes through times. These effects can be examined in both microscopic and submicroscopic scales. Remarkably, the pitting corrosion rate of the sample under study can be examined by roughness parameters. However, the dynamic speckle pattern methodology reveals the cumulative affects including the activities and the frequently fluctuations of the investigated sample altogether.

Figure 3a–c demonstrate the THSP matrices for a sample experiencing the pitting corrosion at 10, 20, and 30 min after the experiment began, respectively. THSPs were constructed by putting the intensity of 800 random pixels over all of 500 speckle patterns recorded at 50 frame rate per minute. The intensity fluctuations of the points stem from the sample surface activity. The distinct bright horizontal lines and the appearance of discontinued lines in the THSPs over time indicate an increase in the activity of the sample. In cases of extremely high activity, the THSP pattern becomes a usual speckle pattern such that the bright lines are not recognizable similar to a random light field.

In order to better evaluate the changes in activities, the samples’ COM matrices at the aforesaid time intervals were calculated. Fig. 4a–c shows the 3D plot of COM matrix of the THSPs of the sample (depicted in Fig. 3a–c), at every 10 min interval after the experiment began. Level of reference and comparison demonstrate i and j intensity levels in Eq. (1), individually. In Fig. 4 two points can be distinguishable: first the spreading points more around the COM main diagonal are observable and the matrix is similar to cloud by passing time, and second the points number including high values of COM grows in longer times. In accordance to the description of COM and THSP, further activities are in relation with higher frequent and more deviations from the diagonal in time unit. Accordingly, the distributions around its original diagonal is in relation to of the homogeneous samples while the appearing of nonzero elements far from the diagonal shows extremely fluctuations being in the sample.

**Figure 8:**
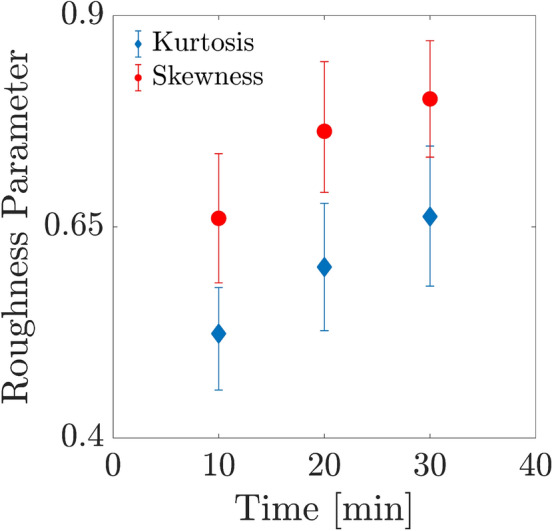
Average skewness and kurtosis of 500 speckle patterns of the sample’s surface as a function of time.

In order to make possible a more quantitative assessing the COM spread values away the principal diagonal such as indicating the activity of the sample, we proceed for computing the IM values of various samples through time. Figure 5 demonstrates the IM value averaged the 10 samples at every 10 min. The description of IM is as summation on the squared row distance respect to the THSP original diagonal. Therefore, it is considered as the quantitative representation of the cloudiness, for example data spreading COM distribution amount away from original diagonal. In Fig. 5, increasing IM values and the activity of the samples is as result of pitting corrosion while time passing through the experiment. Accordingly, error bars are the average of ten IM values for the 10 samples. The IM increase could have attribution to the activity while corrosion pitting.

**Figure 9:**
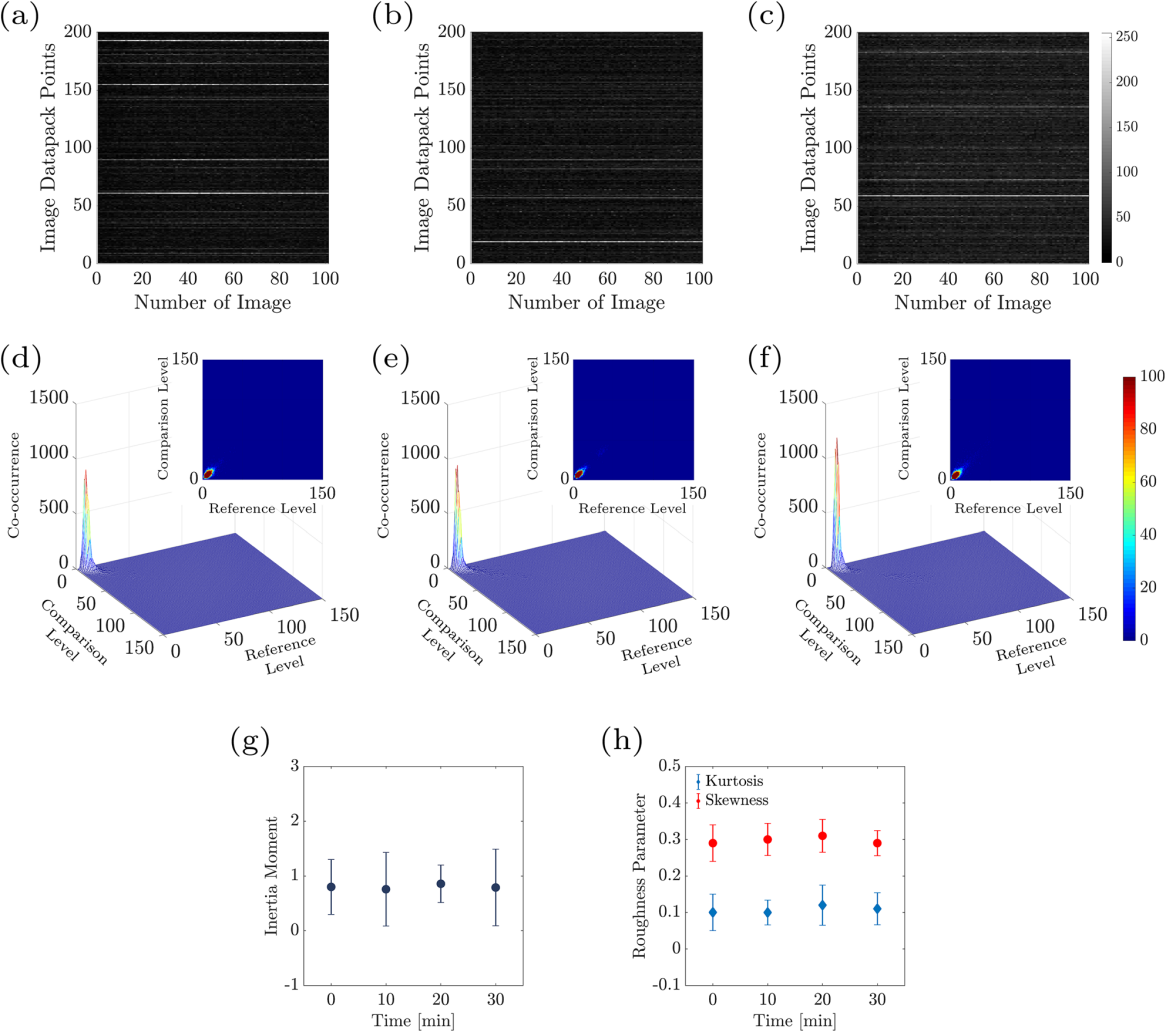
Time history speckle pattern (THSP), formed by tracking 200 random points throughout a collection of 100 speckle patterns, of the sample at (**a**) $\hbox {t}=10 \,\hbox {min}$, (**b**) $\hbox {t}=20\,\hbox {min}$, and (**c**) $\hbox {t}=30 \,\hbox {min}$ after the experiment starts. (**d**–**f**) 3D plot and 2D map of COM matrix associated with the THSP matrices of controlling experiment (**a**–**c**) every 10 min after the experiment starts. Reference level and comparison level show intensity levels of i and j in Eq. (1), respectively. (**g**) Average inetia moment over the THSPs associated with the sample in controlling experiment as a function of time. (**h**) Average kurtosis and skewness of 100 speckle pattern of the controlling experiment as a function of time.

For a separate assessment of the pitting corrosion process the MHI is also considered as speckle parameter for examination of the pitting evolution and sample activities. The MHI parameter is used for image sequences which are recorded at 10, 20, and 30 min by the camera. Figure 6a–c represent analysis related to MHI. Figure 6a–c are indicating the areas activity in pseudo color images resulted from the MHI technique. During 30 min of the sample evolution, there is an effective possibility detectable by following the activity 2D maps. The high change that happened in the activity represented more dots density, it is while less activity is associated with a smaller dots density in the last processed image of MHI. The background is presented in blue as expected. It shows that, as time passes the number and size of the pit are growing and include the higher activity. According to Fig. 6a–c, the evolution of pitting corrosion growth during 30 min can be processed with MHI. So, it is possible to illustrate the temporal growth evolution of pitting corrosion in real-time qualitatively.

It is clear that the surface of pitting is changing over the time, which makes the surface less uniform and causes a increase in the roughness of the sample. In this study we proceed for calculating the sample roughness by the graphical skewness parameter for the speckle pattern. As shown in Fig. 7 we plotted a 2D map of the graphical skewness matrix for exemplary to show the roughness changes and increases through the time very ten minutes as colour bars shows. We examined the roughness of the sample by calculating the kurtosis and skewness parameters of the speckle pattern of the pitting corrosion. These parameters give general information concerning the structural changes of the surface of the sample. For structures similar to what we have investigated here, the information related to the roughness is important. Figure 8 shows the skewness variations and kurtosis parameters. Every data point is acquired by averaging over the skewness of 500 speckle patterns (every 10 min), related to each sample. The error bars are associated with taking the average over the fifty skewness and kurtosis mean values, and of roughness mean values which are calculated by taking average over the 500 speckle patterns recorded for each sample. These parameter changes demonstrate that the samples surfaces become rougher by passing time as the pitting corrosion activities happen in the samples under study. In Fig. 8, the growth in the roughness parameters values in the primary steps is more than the final steps.

It is noteworthy that the roughness parameters of the intensity distribution across the speckle field are calculated and assumed to resemble the roughness of the sample surface. However, toward a comprehensive description of the sample surface, considering different moments of deviations from the average values is required, which are the introduced and reported parameters, i.e. skewness and kurtosis. For example, negative skewness indicates a predominance of valleys, i.e. low intensities, while positive skewness indicates a peaky distribution. A value of $R_{P3}$ = 0 indicates a surface with a symmetric intensity distribution, while $R_{P3}$ > 1 ($R_{P3} < 1$) indicates the presence of extreme peaks (valleys) on the pattern. Kurtosis ($R_{P4}$) is a parameter that measures the sharpness of the distribution across the pattern. For a perfectly random distribution of intensities with a Gaussian probability density function, $R_{P4}$ = 3. Kurtosis is related to the width of the intensity distribution. $R_{P4}$ values smaller than 3 indicate broader distributions corresponding to speckle patterns described as gradually varying, free of extreme peaks or valley features in the intensity distributions. Values greater than 3 indicate the presence of inordinately high peaks or deep valleys. Moreover, as the pitting becomes more pronounced, these values will increase and their number will grow. Since corrosion includes the valley, the absolute amount of it will increase over time, as shown in Fig. 8 of the manuscript.

Moreover, we can assess the rate of corrosion growth based on the rates of skewness and kurtosis. The roughness parameters of the intensity distribution throughout the speckle field have been calculated and assumed to resemble the roughness of the sample surface. Although these parameters are inherently different (the former is associated with intensity fluctuations and the latter with height fluctuations), it is the rough surface that produces the speckle pattern when illuminated with a laser beam due to the scattering of light rays. Similar trends of the two different parameters for various samples have already been studied and reported in references^49,50^. According to the common definition of kurtosis as a degree of peakedness, the difference in distribution width with different kurtosis values is demonstrated in references^51–54^ and it is mathematically proven in^55^.

In order to subtract the applied electrical forces as well as the environment around the sample, we conducted control experiments. The results of a typical control experiment are presented in Fig. 9. In Fig. 9, THSP (a–c), 3D and 2D plot COM (d–f), IM, and roughness parameter are plotted at t = 10, 20, and 30 min. Reference level and comparison level show intensity levels of i and j in Eq. (1), respectively. Figure 9g shows the average inertia moment over the THSPs associated with the sample in the control experiment as a function of time. As shown in Fig. 9h we plotted the average kurtosis and skewness of 100 speckle patterns of the control experiment as a function of time.

Our method of dynamic speckle pattern analysis for pitting corrosion is an online and real-time method in the pitting corrosion growth evolution, including the high-resolution characteristic that is of utmost use for a similar dynamic phenomenon. Most importantly, the experimental assessment as a control has been performed which verifies our results.

## Conclusion

In summary, we presented a novel low-cost method for real-time monitoring of pit initiation and growth in a two-point bending specimen. The experiments were carried out on a Custom 450 stainless steel sample placed in 3.5 wt% NaCl solution. The evaluation of pitting corrosion, including its surface features, was performed based on dynamic speckle pattern analysis. Dynamic growth measurement of pits was obtained through the examination of morphological and various statistical parameters of the surface. Changes in surface roughness showed an increased corrosion-related surface activity. The results showed that the starting point and growth rate of the pits gradually change over time so that new ones are formed each time.

## Data Availability

The datasets used or analysed during the current study available from the corresponding author on reasonable request.
